# General practice as a place to receive help for domestic abuse during the COVID-19 pandemic: a qualitative interview study in England and Wales

**DOI:** 10.3399/BJGP.2022.0528

**Published:** 2023-09-19

**Authors:** Elizabeth Emsley, Caroline Coope, Emma Williamson, Estela Capelas Barbosa, Gene Feder, Eszter Szilassy

**Affiliations:** Bristol Medical School, University of Bristol, Bristol.; Bristol Medical School, University of Bristol, Bristol.; IRISi Interventions, Bristol.; City, University of London, London.; Bristol Medical School, University of Bristol, Bristol.; Bristol Medical School, University of Bristol, Bristol.

**Keywords:** COVID-19, domestic violence, pandemics, primary health care, qualitative research, referral and consultation

## Abstract

**Background:**

General practice is an important place for patients experiencing or perpetrating domestic violence and abuse (DVA), and for their children to seek and receive help. While the incidence of DVA may have increased during the COVID- 19 pandemic, there has been a reduction in DVA identifications and referrals to specialist services from general practice. Concurrently there has been the imposition of lockdown measures and a shift to remote care in general practices in the UK.

**Aim:**

To understand the patient perspective of seeking and receiving help for DVA in general practice during the COVID-19 pandemic. This was then compared with experiences of general practice healthcare professionals.

**Design and setting:**

A qualitative interview study in seven urban general practices in England and Wales, as part of a feasibility study of IRIS+, an integrated primary care DVA system-level training and support intervention.

**Method:**

Semi-structured interviews with 21 patients affected by DVA and 13 general practice healthcare professionals who had received IRIS+ training. Analysis involved a Framework approach.

**Results:**

Patients recounted positive experiences of seeking help for DVA in general practice during the pandemic. However, there have been perceived problems with the availability of general practice and a strong preference for face-to-face consultations, over remote consultations, for the opportunities of non- verbal communication. There were also concerns from healthcare professionals regarding the invisibility of children affected by DVA.

**Conclusion:**

Perspectives of patients and their families affected by DVA should be prioritised in general practice service planning, including during periods of transition and change.

## INTRODUCTION

Domestic violence and abuse (DVA) is a major public health and clinical concern, and has widespread impacts for those affected, the local community, and wider society.^1^ DVA can have multiple health consequences for patients, including physical and mental ill health.^2^ DVA can also impact children, causing significantly harmful short-term and long-term effects.^3^

Being affected by DVA can result in increased health service use: women affected by violence are more likely to contact healthcare services than women who do not face abuse.^4^ Men affected by DVA also present to healthcare settings, including general practice, where men may seek help when experiencing or perpetrating DVA.^5^ Patients presenting in general practice may have a higher lifetime prevalence of DVA, compared with the general population.^6^^–^8 Indeed, patients affected by DVA value being listened to by their healthcare provider as well as the offer of practical support.^9^ Therefore, general practice has an important role in identifying and responding to patients experiencing or perpetrating DVA, and their children. General practice should be part of the multi-agency response to DVA, providing a vital link to specialist DVA support services.^10^ General practice also has an important role in patient follow-up and risk assessment.^11^^,^^12^

While the incidence of DVA may have increased in the COVID-19 pandemic, including an increase in complex and serious cases,^13^^,^^14^ the imposition of severe lockdown measures has occurred at the same time as a significant reduction in general practice DVA identifications and referrals to specialist services.^15^ Help- seeking in primary care has been reduced in other health areas, including mental health and cancer.^16^^,^^17^ General practice has also faced escalating competing demands during the pandemic, with reported challenges in accessing primary care and scheduling appointments.^18^

At this time, there has been a shift to remote general practice consultations, incorporating telephone, video, or online consultation platforms, as an anti-contagion strategy in primary care.^19^ General practices have achieved a rapid and creative transition to remote consulting.^20^ A study involving 21 general practices in South West England indicated a 90% shift to remote consultations by April 2020.^19^ Practices flexibly determined when face-to-face contact was necessary, for example, according to clinical need.^20^ For practice nursing teams, face-to-face contact continued in the form of activities such as blood tests and home visits, with nurses combining multiple tasks at the appointment to optimise the face-to-face contact.^20^

**Table:** How this fits in

General practice is an important place for patients experiencing or perpetrating domestic violence and abuse (DVA) and for their children to seek and receive help. While the incidence of DVA may have increased during the COVID-19 pandemic, there has been a substantial reduction in DVA identifications and referrals to specialist services from general practice. At the same time, there has been the imposition of stringent lockdown measures and a rapid shift to remote care in general practice. This study explored patient experiences of seeking help for DVA in general practice during the COVID-19 pandemic, with additional insight from healthcare professionals. This study also included a focus on children affected by DVA. The authors found that patients affected by DVA had a strong preference for face-to-face consultation models in general practice for the opportunity of non- verbal communication. Children affected by DVA are a vulnerable group and this study reported concerns regarding their visibility to healthcare professionals in general practice during the pandemic.

Online consultations can improve access to care, for example, enhancing convenience, efficiency, and supporting access for those facing verbal communication difficulties.^21^ However, there have been concerns of increased workload for practices and restricted access to care for digitally excluded patients.^21^ Concerns regarding patient safeguarding in remote consultations have also been reported. GPs have worried about missing visual cues during remote consultations and have had patient safety concerns, including in the context of DVA.^22^ Reduced continuity of care has also damaged opportunities for patient safeguarding.^22^

General practice healthcare professionals’ perspectives highlight the importance of maintaining access to primary care, collaborative working, and a whole-team approach, as well as establishing clinical encounters that facilitate a disclosure, such as transitioning from remote to face-to-face consultations, which are crucial in the primary care response.^12^ However, there is a gap in understanding of the patient experience of help-seeking for DVA in primary care during the pandemic and how this compares with the perspectives of healthcare professionals.

During and prior to the pandemic, the authors tested the feasibility of Enhanced Identification and Referral to Improve Safety (IRIS+) in England and Wales. IRIS+ is an integrated primary care system- level training and support intervention, enhancing and extending the original predominantly female adult survivor- focused IRIS model to all patients affected by DVA. The IRIS+ model involves tailored training for non-IRIS-trained and IRIS-trained general practices about DVA, as well as a referral pathway to local specialist DVA support for adult (women and men) survivors, perpetrators, and children. IRIS is a nationally recognised specialist DVA training, support, and referral programme for general practices, favourably evaluated in a randomised controlled trial with demonstrable cost- effectiveness.^23^ IRIS aims to nurture greater health service engagement with DVA by partnering primary care with the third sector specialist domestic abuse services.^24^ Advocate educators are based in specialist domestic abuse services and offer specialist DVA support and expertise in the IRIS programme, with their role including general practice staff training, as well as receiving referrals for patients affected by DVA and offering expert advocacy for these patients.^24^

The emergence of the COVID-19 pandemic during the IRIS+ feasibility study led to an additional key area of investigation on the impact of the pandemic on DVA identification and response in general practice, in the context of the IRIS+ intervention. When comparing pre- pandemic and pandemic time periods in the IRIS+ study, the latter period saw a one-third reduction in referrals from study general practice teams, which corresponds to findings from other studies.^15^ There was also a nearly 80% increase in third- party DVA identifications, such as from the police, who subsequently notified general practices.

In the context of reduced referrals from general practice teams to specialist DVA services, this study aimed to understand the patient perspective of seeking and receiving help for DVA in IRIS+ general practices during the COVID-19 pandemic. These perspectives were compared with experiences of general practice healthcare professionals participating in the IRIS+ intervention who delivered care to those affected by DVA during the pandemic.

## METHOD

In a feasibility and acceptability study for the IRIS+ training intervention, seven general practices were sampled from two areas of England and Wales. The training intervention was developed by a multidisciplinary team,^25^ and involved one or two 2-hour training sessions (depending on previous IRIS training), an additional brief (up to half an hour) remote reminder, and a question and answer session for general practice staff. Training was face-to- face, with the provision of supplementary online resources. Training also included an additional brief online reminder, and a question-and-answer session during a clinical practice meeting. Content of training sessions aimed to enhance the identification and referral of women, men, and children affected by DVA and presenting to general practice to specialist DVA services. The IRIS+ intervention commenced in early June 2019 and ended on 31 December 2020. This substudy focused on the impact of the pandemic on the identification and response to DVA in general practice in the context of the IRIS+ intervention.

### Data collection

The authors interviewed patients affected by DVA including women, men, and children, following their referral from general practices trained by IRIS+ to a specialist DVA service, where they received support as part of the IRIS+ intervention, and/or on completion of specialist support. Patient interviews relevant to this substudy were conducted between April 2020 and August 2021, with this data collection occurring during COVID-19 lockdown periods. Healthcare professionals from general practices participating in the IRIS+ feasibility study included GPs, practice nurses, urgent care practitioners, and allied roles such as drug support workers and health visitors. Semi-structured interviews were conducted during and after the IRIS+ intervention, remotely or face-to-face. The semi-structured interview topic guides were developed from the literature, the authors’ previous research, and with the input of two service user expert groups, including women and men survivors. The topic guides were further adapted as new themes or areas of interest emerged during the course of the study. The authors developed and used different topic guides for patients (adults, children, and young people) and for primary care clinicians. Topic guides are provided in supplementary material (see Supplementary Information S1). The topic guides were used for the wider feasibility study, and the authors only report COVID- 19 pandemic specific aspects of the study in this article, with a focus on patients seeking and receiving help for DVA, as well as the general practice response to DVA. Other areas of focus in interviews with the primary care team included their experience of the IRIS+ intervention. In patient interviews, their experience of the referral process and receiving support was explored. In interviews with adult patients, there was consideration of living situation, employment, and/or access to financial support, as well as context with regards to relationship and children. In interviews with children and young people, their hobbies and school experiences during the pandemic were included in the topic guide.

### Analysis

Interviews were audiorecorded and transcribed verbatim. They were anonymised and analysed using manual coding. Using a Framework approach, a coding frame was iteratively developed by the multidisciplinary team from concepts that emerged from data and through regular discussion, and following recommendations developed from discussions with a panel of service user experts. A matrix of summarised data was produced in Microsoft Excel. This provided a structure to refine the framework and identify key qualitative themes.

## RESULTS

### Sample characteristics

Interviews with 21 patients affected by DVA (11 women, 6 men, and 4 children or adolescents, who were all boys) and 13 general practice healthcare professionals (7 GPs, 3 practice nurses, a drug support worker, urgent care practitioner, and a health visitor) were included in this substudy (Tables 1–3).

**Table 1.** Characteristics of adult participating patients (*n* = 17)

**Characteristic**	***n* (%)**
**Sex**	
Female	11 (65)
Male	6 (35)

**Ethnicity**	
White British	12 (71)
Black British, Caribbean, or African	2 (12)
Asian or Asian British	2 (12)
Not specified	1 (6)

**Age group, years**	
18–24	1 (6)
25–34	2 (12)
35–44	6 (35)
45–54	6 (35)
55–64	1 (6)
≥65	0 (0)
Not specified	1 (6)

**Table 2.** Characteristics of child or adolescent participating patients (*n* = 4)

**Characteristic**	***n* (%)**
**Sex**	
Female	0 (0)
Male	4 (100)

**Age group, years**	
<10	1 (25)
10–15	2 (50)
≥16	1 (25)

**Table 3.** Characteristics of participating healthcare professionals (*n* = 13)

**Characteristic**	***n* (%)**
**Sex**	
Female	9 (69)
Male	4 (31)

**Type of healthcare professional**	
GP	7 (54)
Practice nurse	3 (23)
Urgent care practitioner	1 (8)
Drug support worker	1 (8)
Health visitor	1 (8)

**Years spent working in a primary healthcare setting**	
0–3	4 (31)
4–9	3 (23)
10–20	3 (23)
>20	2 (15)
Not specified	1 (8)

### Findings

The authors compared experiences of patients affected by DVA with the perspectives of general practice healthcare professionals across four key themes: 1) coping with and responding to DVA during the pandemic; 2) availability of general practice; 3) consultations with general practice; and 4) the general practice response to DVA.

#### Coping with and responding to DVA during the pandemic

To provide vital context for patient and general practice healthcare professional interactions, this study shared perspectives from each group on how they experienced the pandemic in general. Interviews with patients affected by DVA helped to explain the health and social circumstances of patients when interacting with general practice. This study compared this with perspectives of healthcare professionals responding to DVA during the pandemic.

a) Adult patients. Adults affected by DVA provided a window into their life during the pandemic and in lockdowns, which for some patients had been traumatic. Those still in abusive relationships experienced worsening abuse.

One patient discussed the impact of the pandemic on his mental health:

*‘*[I] *had to endure what I’ve endured before I’ve managed to escape and have the support which I needed, and then this has all come about now … It’s just almost like a double whammy for me, of the horrors I had to deal with last year, and to make that big step to do it, I have now, and there’s something else now to worry about.’*

(Patient 6, male adult)

While a female patient experienced worsening coercive control and severe restrictions on her independence:

*‘I felt like I couldn’t do anything because I had to be around him all the time.’*

(Patient 9, female adult)

For others, the pandemic presented additional stressors, including financial and housing worries.

b) Child and adolescent patients. Children and adolescents affected by DVA shared information on their social circumstances during the pandemic, with direct relevance to their health and wellbeing.

Children affected by DVA discussed school closures during lockdown, with some feeling that they ‘didn’t mind it’ and one child feeling relieved to be at home:

*‘Because I don’t like* [the virus].*’*

(Patient 18, male child)

However, an adult participant, whose family were affected by DVA, was concerned about his 12-year-old son’s wellbeing at home:

*‘He’s done nothing since February* — *well, since COVID, since leaving school in March, he would have been on that computer over 14 hours every day.’*

(Patient 7, male adult)

An adolescent shared their worry regarding a shortage of services for mental health or relationship advice when they had been affected by DVA:

*‘There’s a lot for little children, but not quite a lot for teenagers who are neither adults or eligible to be children any more. So, pretty hard for us … You’ve got that gap between 16 to 18 where nobody can really help you out with relationships or with your feelings.’*

(Patient 21, male child)

c) Healthcare professionals. While trying to support patients affected by DVA, healthcare professionals also needed to rapidly adjust to new ways of working, including uncertainty regarding their future working practices:

*‘Yes, a very different way of working at the minute, but it seems to be getting back to some type of normality in my other surgeries, but then it’s ongoing, we’re not sure how with the second lockdown or what is going on, forward. So, it’s kind of just being flexible and going with the flow.’*

(Drug support worker)

Although there was a strong motivation to keep DVA in mind, there were competing pressures:

*‘We’re trying desperately to deal with the physical, the COVID that’s going on, the massive mental health that’s going on. We’ve just got to keep remembering and trying not to switch off.’*

(GP 1)

#### Availability of general practice

Patients reported barriers in accessing general practice during the pandemic: from a perception of general practice *‘being shut’*, a fear of contracting the COVID-19 virus when entering the practice, to difficulties in arranging appointments. This occurred at the same time as a national general practice shift to remote consultations, aimed at minimising COVID-19 infection risk.

One patient described occasions of wanting to contact his GP. However, he was stopped by a perception of the practice being closed:

*‘A couple of times I felt like I would have liked to have gone and seen them again, but with it being shut, I think I just left it … It’s basically, with the virus going on, I thought “Well, I’ll just get on with it a bit.” ’*

(Patient 8, male adult)

Some patients discussed challenges in arranging a general practice appointment during the pandemic, for example, when wishing to discuss their medication:

*‘It was just very difficult to get an appointment with the doctor, for them to review me, to continue. And I felt like I needed to almost go up on the medication. And I got to the point where I was like, “Do you know what? It’s causing me more stress trying to get hold of the medication.” ’*

(Patient 15, female adult)

While healthcare professionals acknowledged difficulties in access for those affected by DVA was *‘scary’*, they were being overwhelmed with the widening remit of general practice in response to the pandemic. As one nurse articulated:

*‘That’s the scary thing is, it’s the most vulnerable time and probably the least … they have got the least accessibility to the practice and the GPs.’*

(Practice nurse 3)

#### Consultations with general practice

The authors compared patient experiences of consultations in general practice with those of healthcare professionals, including perspectives on face-to-face versus remote consulting. While there was limited discussion with children about their experiences with general practice, healthcare professionals raised concerns about the visibility of children affected by DVA in remote consultations.

While some patients valued remote consultations when receiving focused long-term support from DVA specialist services, this was not reflected when disclosing to or receiving support from a GP. In a GP consultation, male and female patients overwhelmingly preferred face-to-face consultations to remote consultations. Reasons included being able to see the response of another person being facilitated when discussing personal DVA experiences:

*‘Well, it is just being in the room with someone, isn’t it? And being able to open up to someone, and actually see who you are talking to, and see their responses when you are talking to them about problems.’*

(Patient 9, female adult)

Others valued a face-to-face consultation for the opportunity to express non-verbal communication:

*‘I prefer to see people face-to-face because I prefer to see their body language and for them to see my body language, because I talk a lot with my hands.’*

(Patient 15, female adult)

Healthcare professionals agreed that remote consultations were limited by the loss of non-verbal communication, complicating DVA identification:

*‘We got so much out of seeing our patients and physically seeing all the non-verbal cues. So, the advantage of having a* [face- to-face appointment] *is that I can talk to someone, and I know what they look like, and I know what they sound like, and I can tell when they’re not right.’*

(GP 1)

However, one GP shared a specific case when a video consultation was helpful in achieving face-to-face by proxy:

*‘She was one where I actually really saw the value of doing a video consultation … I spoke to her over the phone and she was kind of holding it together, and then when I spoke to her on video, it was just that, speaking to someone face-to-face where she broke down in tears … ’*

(GP 3)

When discussing children with healthcare professionals, there was concern that an absence of face-to-face appointments had resulted in missed visual cues. Visual cues may be relied on to help identify DVA, especially if children may not communicate problems verbally:

*‘We’re not seeing children, we’re not seeing whether they’re scruffy, unkempt, bruised, we’re not seeing women. All the cues that you would have got before, you’re not getting.’*

(GP 1)

Healthcare professionals also noted that, in face-to-face appointments, they could ask a relative to *‘step out for a minute’*, and remote consultations weakened opportunities to speak to children alone. They also found:

*‘Lots of children tend to not want to speak* [to me] *over the telephone anyway and I end up speaking with their parents.’*

(Urgent care practitioner)

Finally, one GP emphasised that face- to-face consultations had still taken place throughout lockdowns, although they acknowledged differences in communication across consultation models:

*‘It is not that we have ever stopped seeing any patients, even at the height of the pandemic we were seeing patients, but yes, I guess the conversation and the relationship is slightly different when we are not face-to- face.’*

(GP 6)

#### The general practice response to DVA

The authors compared what patients valued in the response from general practice with the strategies used by healthcare professionals to identify and respond to DVA. Patients appreciated a flexible, time- efficient response, as well as continuity of care and signposting to relevant resources. Healthcare professionals found a proactive, intuitive approach was important in consultations, as well as awareness of historical DVA in the medical record, multidisciplinary working, and connectivity between general practice and DVA services.

In receiving help, patients valued a prompt and flexible response from the practice. One patient shared how this response enabled them to speak freely, away from an abusive partner:

*‘It was done over the telephone to the COVID situation. Originally, I contacted her, and my husband was in the room at the time, so I said about some issues that I was experiencing, and the things that I could say in front of him. Then she asked me to pop down to the surgery to get a couple of tests, and at that point I spoke to the receptionist who came out to me. I explained that I needed to hopefully speak to the doctor again. I said, “I want to talk without my husband being around.” She quickly said, “That is absolutely fine, go and sit in the car and we will contact the doctor to call you again.” Within about ten minutes my doctor called me back and I was able to have a confidential conversation without my husband being there.’*

(Patient 10, female adult)

Continuity of care from the same GP was felt to be essential:

*‘She’s been with me all throughout my journey of feeling anxious.’*

(Patient 12, female adult).

In addition, patients appreciated relevant signposting and the availability of options when discussing DVA:

*‘She listened to what I needed to say, and gave me all the options that would be available that I would consider … Yes, it was quite a nice conversation in the grand scheme of things.’*

(Patient 10, female adult)

When trying to identify and respond to patients affected by DVA, healthcare professionals were proactive and used their instinct:

*‘Something that makes you think, “Oh, that doesn’t sound right.” So, you’ve got to be much more switched on … I’ve spoken to one person and said, “Are you safe? Can you talk to me? Are you safe at home?” and that was … I don’t even remember now why I wondered about that, but it just felt something that I needed to check … A lot of it is instinct.’*

(GP 1)

Alerts in the electronic medical record, including if a patient has experienced historical DVA, was another useful reminder for healthcare professionals to think about DVA in consultations. Sharing concerns about patients in multidisciplinary meetings, including with midwives, health visitors, and palliative care teams, was an additional mechanism in identifying and responding to DVA using a team approach.

## DISCUSSION

### Summary

By exploring patient and general practice healthcare professional perspectives, this study highlighted positive experiences of general practice as a place to seek help for DVA during the pandemic. There have also been barriers in accessing general practice and challenges with remote consultation models, during a time of major transition, rapid adaptation, and uncertainty in primary care.

As general practice rapidly transitioned to remote consultations during the COVID- 19 pandemic, patients had a perception of general practice *‘being shut’*, compounded by fears of the virus itself, and healthcare professionals acknowledged that this was a high-risk time for those affected by DVA. When consulting with general practice, patients overwhelmingly preferred face- to-face consultations for the opportunities of non-verbal communication. Healthcare professionals also felt the loss of non-verbal communication in remote consultations made identifying DVA more challenging and they felt particularly concerned regarding the invisibility of children to primary care during the pandemic.

In the general practice response to DVA, patients valued a flexible, time-efficient response, as well as continuity of care. Flags in the electronic patient record about past DVA experience and multidisciplinary information sharing helped GPs to keep DVA in their minds. These alerts may be a pop-up box when clicking on the patient’s electronic medical record or in the medical history section of the record. However, these alerts are on the general practice electronic patient record platform and may not be consistently shared with specialist DVA services, unless added to the referral form by the clinician. Overall, a proactive and intuitive approach by clinicians was important in effectively identifying DVA.

This study also provided valuable context on the health and social circumstances of patients affected by DVA during the pandemic, and general practice professional perspectives of responding to DVA during this time.

### Strengths and limitations

This research offered a variety of perspectives on help-seeking for DVA in general practice during the pandemic, including those of general practice healthcare professionals in diverse roles, female and male patients affected by DVA, and the voices of children. This substudy was developed while the IRIS+ feasibility study was already in progress. However, the authors were able to swiftly adapt the qualitative data collection tools and procedures to facilitate the exploration of the impact of the pandemic on the identification and response to DVA in general practice. Albeit the analysis partially relied on data collected for the wider study. Validity and reliability of the findings were ensured by constant data comparison and comprehensive data use.^26^ A study limitation was that the authors were unable to report the length or type of doctor–patient relationship that patients may have had with their GP or with the GP practice. This may impact on patient preference for face-to-face or remote encounters. Moreover, this study was unable to specify the exact date of DVA disclosure, and whether a patient was experiencing current or historical DVA may affect their preference for a remote or face- to-face encounter. The generalisability of this study’s findings is limited by the study only being performed in the context of the IRIS+ intervention. The general practices had specific guidance and booster training in the context of the COVID-19 pandemic, as well as a referral pathway to a specialist DVA service. Patients interviewed have been identified as being affected by DVA and have also received support as part of the IRIS+ intervention. This study did not capture the perspectives of unidentified and unheard patients who were experiencing DVA during the pandemic and have found themselves without support.

### Comparison with existing literature

There is an emerging literature regarding general practice as a place to seek help during the pandemic in the UK. This study’s findings highlighted barriers in accessing general practice for patients affected by DVA, in line with studies investigating other health conditions.^16^^,^^17^

This study’s findings underline concerns already voiced, including by DVA specialist organisations, regarding the loss of non- verbal cues in remote consultations and the impact this has on identifying DVA.^27^ Others have also highlighted safeguarding concerns more generally in remote consultations.^22^ Some studies reflect that online general practice consultations can favour simple, transactional clinical encounters.^21^ However, this study’s findings emphasised that, for some patients affected by DVA, a face-to-face general practice consultation allows them to safely share sensitive and complex information using body language.

While this study’s findings indicated a strong preference among patients affected by DVA for face-to-face general practice appointments, some patients did value remote encounters in the context of support from specialist DVA services. The reason for this change in patient preference across two different contexts is unclear and more research is needed. Potential explanations may include that DVA is already identified by the time patients receive support from DVA services, compared with when they attend a GP appointment for an initial disclosure. Patients being willing to have remote DVA specialist support has been echoed in other literature.^27^

Research focusing on the health care of children affected by DVA in general practice during the pandemic is limited. Parental concern regarding children’s wellbeing at home was reported here, supporting general worries about children’s learning at home during the pandemic.^28^ Concerns regarding the invisibility of children living with DVA, reflected by healthcare professionals in this study, have been raised previously.^29^ The worry of having ‘no eyes on children’, was also raised in a study sharing perspectives of Australian practitioners working remotely in DVA services.^29^ Healthcare professionals can rely on visual assessment and non-verbal cues from children, especially since their verbal articulation of a problem may be different from that of adults. Considering safeguarding of children generally, remote consultations in primary care during the pandemic have damaged opportunities to see the dynamics between parents and children, and complicated identification of ‘red flags’ compared with face-to-face consultations.^22^ This is especially important given avenues to safeguard children were further impacted by concomitant school closures during lockdown periods.^30^

### Implications for practice

By exploring the patient perspective of seeking help for DVA in general practice during the pandemic, this article emphasises the importance of listening to service users during periods of transition and change. Maintaining access to primary care is crucial for those affected by DVA, including during the COVID-19 pandemic. A rapid, proactive, and flexible response by general practice can help to identify and respond to DVA. This study’s findings indicate a strong preference for face- to- face consultations and continuity of care in general practice for patients affected by DVA. The authors recommend general practice teams consider this when planning services informed by patient needs. Primary care support for those affected by DVA can be optimised with continuity of care, multidisciplinary collaboration, and the strengthening of training, support, and referral pathways between general practice and specialist DVA services. Children affected by DVA are a vulnerable group, including during the pandemic, and strategies to support them in primary care are essential.
